# Celebrating Women in Science: Pioneering Contributions to Animal Behaviour and Welfare

**DOI:** 10.3390/ani14081184

**Published:** 2024-04-15

**Authors:** Vera Baumans, Ismene A. Dontas, Pascalle L. P. Van Loo

**Affiliations:** 1 Division Laboratory Animal Science, Department of Animals, Science and Society, Utrecht University, 3584 Utrecht, The Netherlands; 2 Laboratory for Research of the Musculoskeletal System, School of Medicine, National & Kapodistrian University of Athens, 14561 Athens, Greece; idontas@med.uoa.gr; 3 Animal Welfare Body Utrecht, Utrecht University, 3584 Utrecht, The Netherlands; p.l.p.vanloo@uu.nl

In the scientific domain, women have historically demonstrated their dedication, intellect, and innovative input in relation to animal behaviour and welfare. As we delve into the literature, we cannot ignore the impact that women scientists have had on understanding and improving the lives of animals. From groundbreaking discoveries to pioneering improvements, women have carved their names into the annals of history as advocates for animals and their well-being. Historically, women in science have faced barriers and glass ceilings, with their contributions often overlooked. However, their resoluteness has shown to be unshakable, and their work continues to contribute to our understanding of animal behaviour and welfare.

The most famous example is Jane Goodall, whose groundbreaking research on chimpanzees revolutionized our understanding of primate behaviour. Her meticulous observations, conducted over decades at Gombe Stream National Park, not only revealed the complex social dynamics of chimpanzee communities but also underscored the importance of conserving these remarkable creatures. Goodall’s work paved the way for the study of animal emotions, cognitive abilities, and the ethics of wildlife conservation [1].

Dian Fossey, another legendary woman in science, dedicated her life to the study and protection of mountain gorillas in Rwanda. Her work brought to light the threats facing these magnificent animals and catalysed global efforts to safeguard their habitats. Fossey’s story reminds us that science is not only about data and discoveries, but also personal sacrifice and continuous commitment to a cause [2].

The third woman to study under Dr Louis Leakey, the renowned anthropologist, was Birute Galdikas, a Canadian primatologist and ethologist who studied orangutans in Borneo [3]. It was the work of these three women together, called by Leakey “The Trimates”, that changed the way of understanding non-human primates.

With regard to animals in our care, Temple Grandin has shown to be a visionary pioneer, turning the barriers she faced due to autism into her greatest strength. Her innovative designs for livestock handling facilities have greatly improved the meat industry, making it more humane for animals and safer for workers. Grandin’s contributions continue to form and reform animal welfare standards, ensuring that animals raised for food are treated with dignity and compassion [4].

But animals that do not appeal to the human sense of protection can also rely on the dedication of female scientists. Jane Hurst investigates the chemosignalling between mammals in depth and applies this knowledge for the development of humane control of rodent pests [5]. During her work, she discovered the negative effects of the widely dispersed practice of tail handling mice in the laboratory on the welfare of these little critters. The non-invasive handling methods she developed are currently spreading like an oil-slick through the scientific community, positively affecting the lives of millions of laboratory mice around the globe [6].

In addition to these well-known figures, many other women researchers, biologists, veterinarians, and animal advocates are making invaluable contributions to the field. Their work spans diverse areas, some of which have been collected in this Women’s Special Issue Series on Animal Behaviour and Welfare. Contributions range from the latest insights into handling and training wild animals in zoos to exploring gaps in current legislation for animals used in research, and from tackling welfare issues in pets to mapping the habitat needs of wild animals. A common denominator in all these papers is the growing awareness of and urgent call for treating all animals in our care with respect.

The article by Griffin and colleagues [7] presents us with a novel objective framework of shelter and recently rehomed dogs’ needs regarding their quality of life and overall welfare. The authors, after reviewing relevant publications by canine experts, set a prioritization of their needs, grouping related needs and listing them by their nature and by hierarchy as physiological, safety, social, movement, and cognitive needs. Although originally developed for shelter/rehomed dogs, this is a comprehensive framework for the needs of dogs living in a wide range of environments, such as community-owned, working, laboratory, or stray dogs. Importantly, it will allow humans to meet dogs’ needs more accurately, thereby improving dogs’ quality of life and overall welfare.

The next article by Van Leuffen and colleagues [8] deals with broiler welfare by proposing an original solution to increase their activity within their pen; this behaviour can combat poor leg health and body lesions due to inactivity. They scattered dried black soldier fly larvae in broiler pens at different feeding periods and, via camera data collection, observed that this provision stimulated broiler activity and exercise throughout the day and across weeks, contributing to their welfare.

In the following article, Van Loo and Janssens [9] explore why it is ethical to rehome all healthy laboratory animals after the experiments have finished, and report their experience from successfully rehoming more than 1700 laboratory animals over the last four years. In order to ensure the animals’ welfare, they built a framework and provided adoptive owners with recommendations. This offers a welcome continuation of life for healthy animals previously used in research, as proposed by the Directive 2010/63/EU.

The next article by Marinou and Dontas [10] presents the provisions of legislative and guidance documents on the protection of animals used for scientific purposes regarding their welfare, and indicates some areas that need clarification while suggesting potential solutions. Further elucidation on the education and training of persons involved in animal experiments, the quality of retrospective assessments and non-technical project summaries, as well as definitions of specific studies that may or may not be considered as procedures according to the Directive, for example, will be beneficial to laboratory animal welfare.

Gandia and colleagues [11] closely observed giant pandas in an enclosed habitat. They correlated specific cycles of feeding anticipatory behaviour, sexual-related behaviour, and abnormal behaviour with the cycles of the migratory behaviour of their wild counterparts. Their study clearly shows how a holistic approach to animal behaviour and needs using circadian and circannual rhythms gains more detailed and complex information that can be used to adapt the care of captive animals to the time of day, the season, and the age of the animals.

The importance of circadian and circannual rhythms also emerges in the article by Seremak and colleagues [12], who studied the effect of day length and season on mating behaviour in farmed mink. Their study shows the wealth of wild behaviour that is still present in captive mink, and sheds light on the extent to which artificially created breeding conditions infringe on this.

Having spent several years observing Taiwanese leopard cats, Van der Meer and colleagues [13] collected a wealth of information on this animal species that is known to be so difficult to track. They mapped the resting and hunting areas of the cats and showed that these animals prefer to stay hidden and actively avoid areas of human inhabitancy. This research is extremely important in the conservation of this endangered species.

Van Zeeland and colleagues [14] dive into the concept of contrafreeloading and what it means for the well-being of captive grey parrots. They describe the feather-damaging behaviour that is well known in captive parrots restricted in their natural behaviour and compare the motivation to work for food between healthy and feather-damaged birds. Their study reveals that feather-damaged birds that prefer to work for food had most improved plumage, indicating the importance of providing captive parrots with foraging devices.

The invaluable contribution of handling and training to increase the welfare of animals in our care is passionately advocated by Brando and Norman [15]. Their review describes how predictability, controllability, and choice positively affect animal well-being and how positive human–animal relationships are to the mutual benefit of both the animal and caregiver. They highlight best practices in different areas where animals depend on human care and interaction and relate how their findings may affect the numerous animal species in zoos and aquaria.

In conclusion, the Women’s Special Issue Series on Animal Behaviour and Welfare is a testament to the extraordinary contributions of women in science. These brilliant minds have unravelled the mysteries of the animal world, advocated for the ethical treatment of animals, and inspired generations of scientists to follow in their footsteps. Let us celebrate these women and continue to support and empower women in science, ensuring that their vital contributions to the betterment of animal lives are recognized and honoured by future generations.
